# The emergency events database, EM-DAT, must be preserved as a global public good

**DOI:** 10.3389/ijph.2026.1609990

**Published:** 2026-06-15

**Authors:** Niko Speybroeck, Ilan Noy, Albert Kettner, Regina Below, Gabriele Messori, Wim Thiery, Valentin Wathelet, Aglaé Jézéquel, Damien Delforge, Dewald Van Niekerk

**Affiliations:** 1 Institute of Health and Society (IRSS), UCLouvain, Brussels, Belgium; 2 School of Economics and Finance, Victoria University of Wellington, Wellington, New Zealand; 3 Institute of Arctic and Alpine Research (INSTAAR), University of Colorado, Boulder, CO, United States; 4 Department of Earth Sciences and Swedish Centre for Impacts of Climate Extremes (climes), Uppsala University, Uppsala, Sweden; 5 Department of Water and Climate, University of Brussels, Brussels, Belgium; 6 LMD-IPSL, ENS, Université PSL, École Polytechnique, Institut Polytechnique de Paris, Sorbonne Université, CNRS, Paris, France; 7 Ecole des Ponts, Marne-la-Vallée, France; 8 Unit for Environmental Sciences and Management, North-West University, Potchefstroom, South Africa

**Keywords:** disaster databases, disaster epidemiology, disaster risk reduction, EM-DAT, public health preparedness

Disasters are remembered in many ways: through memorials, damaged landscapes, displaced communities, insurance records, and political inquiries. In public health, they are also remembered as data. The Emergency Events Database (EM-DAT), maintained by the Centre for Research on the Epidemiology of Disasters (CRED) at the University of Louvain (UCLouvain), has become part of this global memory. Launched in 1988 as a joint CRED–World Health Organization initiative, it compiles disaster occurrence and human and economic impact data from 1900 onwards, with systematic recording since 1988 [1]. Entries follow transparent inclusion criteria and are curated through daily review of governmental, United Nations (UN), humanitarian, insurance, and media sources [1]. This work is rarely visible: monitoring, coding, checking, refining definitions, and protecting comparability across decades. Yet when such infrastructure weakens, the loss is not merely administrative; it is epistemic. Societies lose part of their capacity to learn from past harm.

**FIGURE 1:**
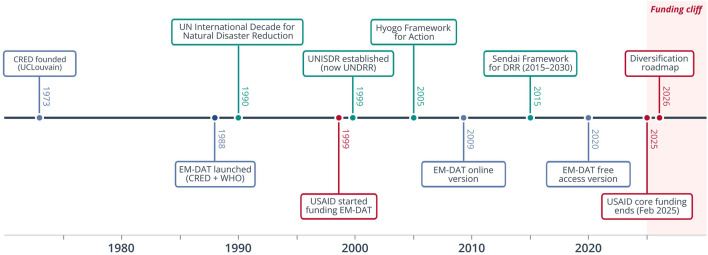
EM-DAT (Emergency Events Database) milestones, 1973–2026. Blue denotes CRED/EM-DAT milestones; red funding milestones; green global disaster-governance milestones. The red shaded area highlights the current funding cliff. Abbreviations: CRED, Centre for Research on the Epidemiology of Disasters; EM-DAT, Emergency Events Database; UCLouvain, University of Louvain; WHO, World Health Organization; UN, United Nations; UNDRR, United Nations Office for Disaster Risk Reduction; UNISDR, United Nations International Strategy for Disaster Reduction; DRR, disaster risk reduction; USAID, United States Agency for International Development; Feb, February (The EM-DAT project, world, 1900-2026).

In early 2025, the dismantling of the United States Agency for International Development (USAID) ended more than two decades of core funding for EM-DAT (Figure 1). This should not be read narrowly as a story about USAID, although the loss of a grant sustaining EM-DAT’s core work is consequential. It is a warning about a broader weakness in global health governance. We ask databases such as EM-DAT to behave like public goods, open enough for researchers, trusted enough for policy, standardized enough for comparison, and responsive enough for crises, yet finance them like temporary projects. A global disaster database cannot be switched on and off according to donor cycles without damaging the continuity that makes it valuable.

Recent scholarship in the International Journal of Public Health (IJPH) shows why this continuity matters. The IJPH editorial on the 2024 Spain floods used EM-DAT to place a contemporary catastrophe within Europe’s longer record of deadly floods, linking remembrance to resilience [2]. More broadly, planetary health has emerged as an important perspective in public health, reflecting growing concern about the health impacts of climate and environmental hazards [3]. A review of extreme weather in the United Kingdom found evidence on physical health, mental health, and socioeconomic impacts, while calling for stronger evidence on economic costs and resilience [4]. A meta-review of heat-related diseases showed that heat affects far more than classical heat illness, extending to cardiovascular, kidney, respiratory, mental health, and infectious conditions [5]. These articles depend on comparing hazards, impacts, and vulnerabilities across time and place.

The same pattern is visible in research on post-disaster services and preparedness. Drawing on lessons from Haiti, Cénat and Derivois proposed evidence-informed measures to protect mental health after the 2023 Türkiye–Syria earthquakes [6]. Sahan et al., in a study of public health professionals after the 2023 Kahramanmaraş earthquake, documented stress, burnout, housing insecurity, coordination challenges, unclear roles, and insufficient resources [7]. Akthar and Reid used the 2022 Pakistan floods to argue for climate-resilient health systems [8]. Such studies provide essential local evidence on disaster impacts and response needs. Their broader public health value, however, depends on the ability to situate individual events within a harmonized global record. Without such a reference point, evidence risks becoming increasingly local, episodic, and fragmented.

The argument is not that every disaster-health study must begin with EM-DAT, or that global counts should replace national surveillance, cohort studies, ethnography, or community testimony. Rather, a shared disaster record gives those sources a common frame. It helps researchers distinguish exceptional events from recurring patterns, allows governments to compare preparedness needs with peer countries, and lets journalists and civil society challenge claims made after crises. It also supports teaching: students see how floods, heatwaves, earthquakes, wildfires, and epidemics are counted, where data are missing, and why uncertainty is part of responsible interpretation. These are public-good functions, not merely technical services.

EM-DAT is not perfect, but that is precisely why it must be sustained. The methodological overview by Delforge and colleagues openly catalogues its known biases, such as temporal reporting effects, under-representation of slow-onset hazards, and gaps in heatwave mortality, so users can interpret the data responsibly [1]. Disaster databases are inevitably shaped by reporting systems, political visibility, media attention, economic valuation, and unequal national capacities to document losses. Heatwaves, indirect mortality, compound events, and small or slow-onset disasters remain particularly difficult to capture. At the same time, the growing methodological literature, including work on criteria for archiving epidemics as disasters, illustrates how EM-DAT can evolve in response to emerging threats [9]. Limitations are not an argument for neglect but for investment. Databases improve through continuity: refining methods, expanding partnerships, and making limitations visible rather than hidden.

The United Nations’ Sendai Framework places “understanding disaster risk” at the foundation of disaster risk reduction [10]. This requires more than hazard maps or mortality counts. It requires longitudinal, comparable, transparent, and accessible evidence on who is affected, how, where losses occur, and how impacts change as exposure, vulnerability, climate, and health systems evolve. EM-DAT cannot answer every question; no single database can. But it provides a common reference point from which countries, researchers, humanitarian actors, journalists, and civil society can begin asking better questions, and ultimately collecting better data.

The policy response to EM-DAT’s current funding shortfall should therefore be practical. First, EM-DAT’s core functions should be funded as global public infrastructure, not as a discretionary add-on to short-term projects. A diversified consortium of governments, multilateral organizations, philanthropic foundations, universities, humanitarian agencies, and responsible private-sector users would be more resilient than dependence on a single major donor. Second, governance should protect scientific independence, transparency, and equitable representation, especially for regions with the largest data gaps [11]. Third, investment should target known weaknesses: subnational geocoding, compound and cascading events, heatwave mortality, indirect health impacts, economic-loss comparability, and regional data hubs. Fourth, open non-commercial access should remain central. Commercial services may help cross-subsidize sustainability, but the core dataset should not drift behind paywalls that exclude the communities most affected by disaster risk.

Funders should also value maintenance. Scientific culture rewards novelty, dashboards, and pilot projects more readily than the patient work of updating records, reconciling sources, documenting changes, and answering user questions. Yet durable public health evidence is built on exactly that labor. The principles governing investments in pathogen surveillance, vital registration, and climate data should apply to disaster-loss data: stable financing, transparent methods, interoperable standards, trained staff, and accountability to users. Treating EM-DAT as vital infrastructure would not freeze it; it would create conditions for improvement.

The cost of maintaining EM-DAT is small compared with the costs of disaster response, reconstruction, and unpreparedness. The real danger is not only that a database might shrink or disappear, but that the world becomes more dependent on partial, proprietary, or incompatible records just as climate change, displacement, urbanization, conflict, and fragility make disaster risk more complex. If the common memory of disasters fragments, so too does the evidence base for prevention.

When disaster data loses its lifeline, the victims are not only researchers. Humanitarian planners lose a trusted baseline. Health systems lose lessons from earlier successes and failures. Ministries lose comparative evidence for preparedness. Communities lose visibility in the global record. Public health should therefore argue not merely to save a database, but to recognize disaster data as critical health infrastructure. EM-DAT has been sustained through patience, expertise, and public-good commitment. Its future requires funding that matches its mission: stable, collective, transparent, and global.
